# Enzymatic synthesis of α-flavone glucoside via regioselective transglucosylation by amylosucrase from *Deinococcus geothermalis*

**DOI:** 10.1371/journal.pone.0207466

**Published:** 2018-11-19

**Authors:** Se-Won Jang, Chi Heung Cho, Young-Sung Jung, Chansu Rha, Tae-Gyu Nam, Dae-Ok Kim, Yeong-Geun Lee, Nam-In Baek, Cheon-Seok Park, Byung-Hoo Lee, So-Young Lee, Hee Soon Shin, Dong-Ho Seo

**Affiliations:** 1 Research Group of Healthcare, Korea Food Research Institute, Wanju, Republic of Korea; 2 Department of Food Science and Biotechnology, College of BioNano Technology, Gachon University, Seongnam, Republic of Korea; 3 Research Group of Industrial Technology, World Institute of Kimchi, Gwangju, Republic of Korea; 4 Graduate School of Biotechnology and Institute of Life Science and Resources, Kyung Hee University, Yongin, Republic of Korea; Wageningen Universiteit, NETHERLANDS

## Abstract

α-Flavone glycosides have beneficial properties for applications in the pharmaceutical, cosmetic, and food industries. However, their chemical syntheses are often limited by a low efficiency or scarcity of substrates. In this study, α-flavone glucosides were enzymatically synthesized by amylosucrase from *Deinococcus geothermalis* (DGAS) using sucrose and various flavones as a donor for glucosyl units and acceptors, respectively. Luteolin was the most effective acceptor in the transglucosylation reaction using DGAS among nine flavone materials (apigenin, chrysin, 6,7-dihydroxyflavone, homoorientin, 7-hydroxyflavone, isorhoifolin, luteolin, luteolin-3′,7-diglucoside, and orientin). The highest production yield of luteolin glucoside was 86%, with a 7:1 molar ratio of donor to acceptor molecules, in 50 mM Tris-HCl buffer (pH 7) at 37°C for 24 h using 2 U of DGAS. The synthesized luteolin glucoside was identified as luteolin-4′-*O*-α-D-glucopyranoside with a glucose molecule linked to the C-4′ position on the B-ring of luteolin via an α-glucosidic bond, as determined by ^1^H and ^13^C nuclear magnetic resonance. This result clearly confirmed that the glucosylated luteolin was successfully synthesized by DGAS and it can be applied as a functional ingredient. Furthermore, this approach using DGAS has the potential to be utilized for the synthesis of various glucosylated products using different types of polyphenols to enhance their functionalities.

## Introduction

Flavones are a type of flavonoid with a backbone of 2-phenylchromen-4-one (2-phenyl-1-benzopyran-4-one); they are plant secondary metabolites with anti-inflammatory, anti-tumor, and anti-cancer effects on the human physiology [1–3]. However, the poor solubility and stability of flavone compounds limit their applications as functional materials in the pharmaceutical, cosmetic, and food industries [3–6]. Some flavones are presented in the form of β-linked sugars (β-flavone glycosides), which have higher solubility and stability compared to the aglycone form [7], and various studies have attempted to synthesize various types of flavone glycosides by transferring sugars to the aglycone form. For example, apigenin-7-*O*-β-glucoside can be effectively synthesized using glycosyltransferase from *Bacillus cereus* (BcGT) [8]. Baicalein-4′-*O*-β-glycoside (β-BG) and luteolin-4′-*O*-β-glycoside (β-LG) also can be produced by glycosynthases with comparably high yields. The conversion yield of flavonoid glycosides (β-BG and β-LG) is 72–95% with 10 mM lactosyl fluoride as a donor and 10 mM flavonoids (baicalein and luteolin) as acceptor molecules in 1 mM Tris-HCl (pH 7.8) at 37°C for 12 h using E197S mutants of the *Humicola insolens* Cel7B enzyme [9]. Kim *et al*. reported that baicalein-6-*O*-α-glucoside (α-BG) can be enzymatically synthesized; the maximum yield of α-BG is 59.1% with 20 mM baicalein and 40 mM sucrose as acceptor and donor molecules, respectively, in 50 mM Tris-HCl buffer (pH 8) at 30°C for 12 h using amylosucrase (1 mg/mL), and the water solubility and absorption rate of α-BG are substantially greater than those of baicalein [10].

Glucosyltransferases (GTase, E.C. 2.4.5.1) and glycoside hydrolases (GHase, E.C. 3.2.1.X) are widely used for transglucosylation [11, 12]. Though GTases have a high bioconversion efficiency, they are mainly used in the pharmaceutical industry owing to the need for expensive substrates, such as UDP-glucose. GHases use low-cost substrates, such as starches and oligosaccharides, for transglucosylation reactions, but they have a low reaction efficiency [13]. Glucansucrase belongs to GHase family 13 or 70, and sucrose, a relatively inexpensive substrate compared to UDP-glucose, can be used as a donor to transfer glucose moiety to various substances [14]. The only enzyme belonging to GHase family 13 is amylosucrase (ASase, E.C 2.4.1.4), which undergoes a transglucosylation reaction by a non-processive mechanism [15]. Several studies have shown that ASase is a more potent glycoconjugating enzyme than GHase 70 enzymes [16, 17]. ASase is able to transfer glucose to phenolic/poly-phenolic compounds (e.g., esculetin, baicalein, catechin, phloretin, rutin, hydroquinone, and vanillin) as receptors as well as glucoside compounds (arbutin, aesculin, piceid, and salicin), and the conversion efficiency is substantially increased when the concentration of the donor is higher than that of the acceptor [10, 18–21]. ASase from *Deinococcus geothermalis* (DGAS) has the highest thermal stability and specific activity among AS from various species, and the glucosylation reaction has been evaluated using various substances [22]. The yield of (+)-catechin transglycosylation caused by DGAS is higher than 97% using catechin and sucrose as an acceptor and donor, respectively [17]. A maximum bioconversion yield of α-arbutin of 90% was obtained at a 10:1 molar ratio of donor (sucrose) and acceptor (hydroquinone) molecules in the presence of 0.2 mM ascorbic acid (used to prevent oxidation of the reaction mixture) [16].

Various flavone materials are available as potential pharmaceutical compounds, but their application is limited due to their low solubility [23, 24]. The purpose of this study was to synthesize flavone glycosides using DGAS. Therefore, we screened for the most efficient acceptor among nine flavone materials and found that DGAS converted luteolin to luteolin glucoside (LG) in high yield. Consequently, we optimized the transglucosylation reaction on luteolin and identified the structure of LG.

## Materials and methods

### Chemicals and reagents

Nine flavone materials (apigenin, chrysin, 6,7-dihydroxyflavone, homoorientin, 7-hydroxyflavone, isorhoifolin, luteolin, luteolin-3′,7-diglucoside, and orientin; each prepared with 2 mg/mL DMSO) were purchased from Enzo Life Sciences (Farmingdale, NY, USA). The recombinant DGAS was prepared according to previous methods [25]. All other chemicals used in this study were of analytical reagent grade and were obtained from Sigma-Aldrich (Merck, Darmstadt, Germany).

### Assay of DGAS activity

DGAS activity was measured by the 3,5-dinitrosalicylic acid (DNS) method. The reaction solution was prepared with 90 μL of 200 mM sucrose in 50 mM Tris-HCl buffer (pH 8), and the reaction was started by adding 10 μL of purified DGAS. After the enzyme reaction at 37°C for 10 min, 300 μL of DNS solution was immediately added and boiled for 5 min. The absorbance of the reaction solution was measured at 545 nm using an EPOCH microplate spectrophotometer (BioTek, Winooski, VT, USA). The fructose concentration in the reaction mixture was calculated using fructose as a standard. The specific activity was defined as the amount of enzyme needed to hydrolyze sucrose to 1 μmol fructose per min in the reaction conditions.

### Dimethyl sulfoxide (DMSO) stability of DGAS

The DMSO stability of the purified DGAS was observed by preincubation in the absence of the substrate using 10%, 20%, 30%, 40% and 50% (v/v) DMSO. After 3 h, the residual activity was measured under standard assay conditions.

### Screening for flavone acceptors

The reaction solutions of nine flavone materials (each 2 mg/mL) were mixed with 100 mM sucrose, 50 mM Tris-HCl (pH 8), distilled water, and DGAS (1.6 U/mL), and incubated at 37°C for 24 h. After the reaction, all reaction mixtures were boiled for inactivation of the enzyme and analyzed by HPLC to identify the synthesis of the transglucosylated form. The percentage of conversion was calculated by the following equation:

$$Conversion(\%)=\frac{{\left[acceptor\right]}_{C}-{\left[acceptor\right]}_{R}}{{\left[acceptor\right]}_{C}}\times 100$$

where [*acceptor*]_C_ is the concentration of the initial acceptor molecule in the reaction mixture and [*acceptor*]_R_ is the concentration of the residual acceptor molecule in the reaction mixture.

### Preparation of LGs from the enzymatic reaction solution

To purify LGs, the reaction was performed using a sample volume of 100 mL. The synthesized LGs were purified using the YMC ODS AQ-HG column (120 Å, 10 μm, 20 × 250 mm) (YMC, Kyoto, Japan) connected to a photodiode array detector by modifying the ÄKTA purifier system (GE Healthcare, Stockholm, Sweden). The purity of fractions was evaluated by HPLC. Selected fractions were evaporated using the Hei-VAP Rotary Evaporator (Heidolph, Schwabach, Germany) and then dried using FreeZone freeze dryers (Labconco, Kansas City, MO, USA). Dried samples were maintained at -20°C until further analysis.

### Analysis of LGs by HPLC and mass spectrometry

The liquid fractions of samples were diluted in a mixture of DMSO and methanol (10:90, v/v) and analyzed using the Alliance HPLC with the 2998 photodiode array detector (Waters, Milford, MA, USA) and a Poroshell 120 SB-C18 column (120 Å, 2.7 μm, 4.6 × 150 mm) (Agilent, Santa Clara, CA, USA). Gradient elution was performed using water with 0.1% formic acid (solvent A) and acetonitrile with 0.1% formic acid (solvent B). Dried samples were dissolved in 10% DMSO in methanol, filtered through a 0.45-μm PTFE syringe filter, and analyzed by Alliance HPLC equipped with a UV detector and mass detector (Waters) to obtain mass spectra (S2C Fig). The peaks were monitored by absorbance at 254 nm and electrospray ionization. Full-scan data were acquired with a mass range of 200 to 1150 *m/z* (centroid) in negative mode.

### Optimization of LG production

Transglucosylation reactions on luteolin were performed with a final concentration of 1.4 mM luteolin (0.4 mg/mL in 10% DMSO) and 10.00 mM sucrose as acceptor and donor molecules, respectively, in 50 mM Tris-HCl buffer (pH 8) at 37°C for 24 h using various activity units of DGAS. All reaction mixtures were filtered through a 0.45-μm syringe filter and analyzed using a Jasco HPLC/UV-Vis system (Jasco Inc., Tokyo, Japan). YMC-Triart C 18 (4.6 mm × 150 mm; YMC, Co., Ltd., Kyoto, Japan) was used for the analysis. The percentage of conversion for transglucosylated products was calculated by peak area normalization.

### Structural analysis of LG by nuclear magnetic resonance (NMR)

The purified compounds (10mg) were dissolved in C_5_D_5_N (500μL) and dispensed in NMR tubes (5mm). The ^1^H and ^13^C NMR spectra for purified LG were obtained using a Varian Inova AS 600 MHz NMR spectrometer (Varian, Palo Alto, CA, USA). The sample was dissolved in C_5_D_5_N at 24°C with tetramethylsilane as an internal standard. The NMR results are described in the supplementary materials (S1 Table).

## Results and discussion

### Acceptor specificity of DGAS using flavones

**Fig 1:**
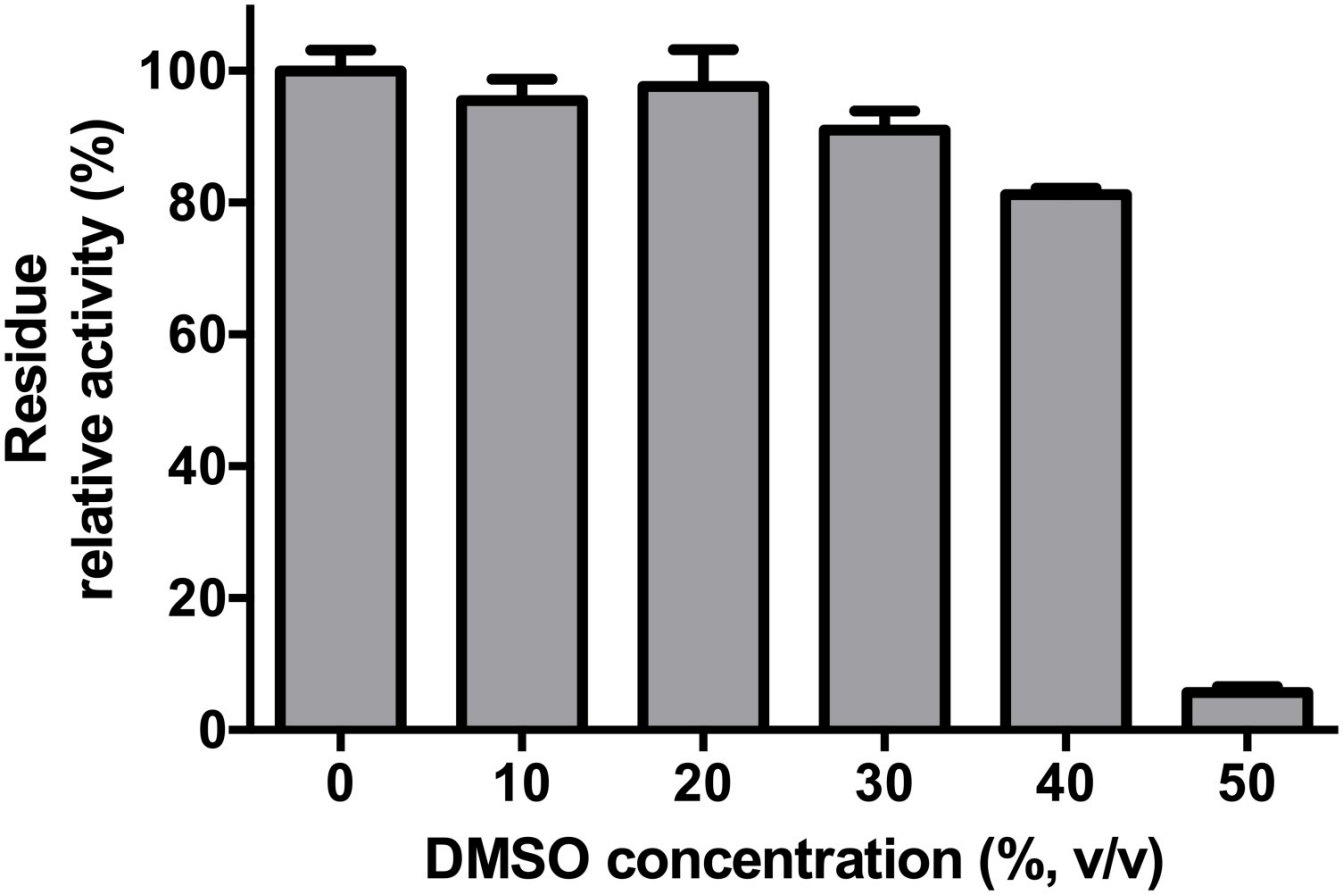
Dimethyl sulfoxide stability of DGAS.

Flavone substances are hardly soluble in water, while they dissolve in organic solvents such as ethanol and DMSO [26, 27]. To confirm the acceptor specificity of DGAS, the different flavone materials were dissolved in DMSO (2 mg/mL), and therefore, DMSO was added to the enzyme reaction solution. As ASase has a low stability in organic solvents and does not show enzymatic activity [28], we tested the stability of DGAS with different concentrations of DMSO (Fig 1). The activity of DGAS was maintained above 95% at 20% (v/v) DMSO concentration and decreased when DMSO concentration exceeded 30% (v/v). In particular, the enzyme activity was decreased dramatically above 50% (v/v) of DMSO. Thus, the 20% of DMSO concentration for DGAS reaction was used.

**Fig 2:**
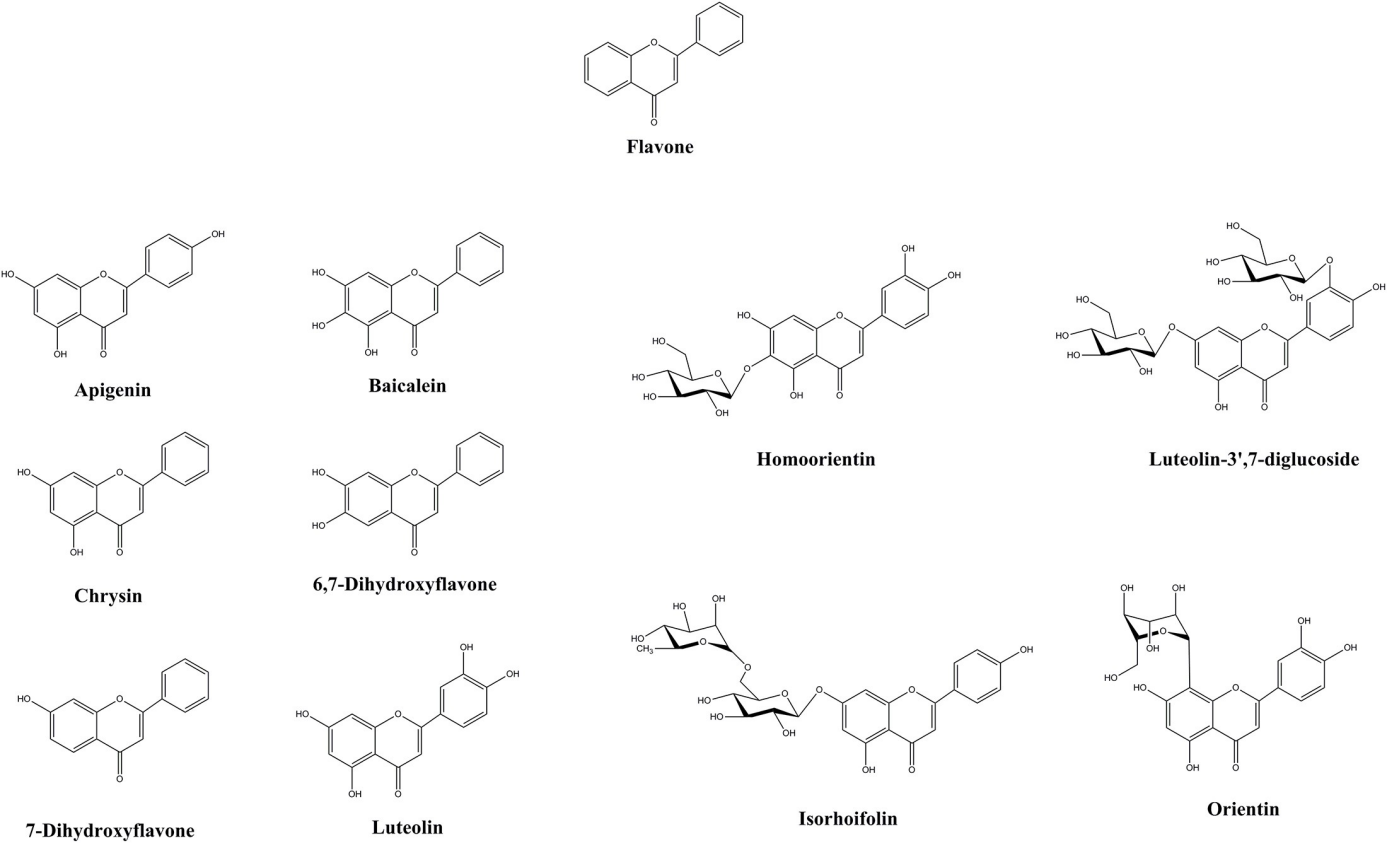
Structures of ten flavones. Structures of the flavone backbone, apigenin, chrysin, 6,7-dihydroxyflavone, homoorientin, 7-hydroxyflavone, isorhoifolin, luteolin, luteolin-3′,7-diglucoside, and orientin are shown.

ASases transfer glucose from sucrose as a donor to the hydroxyl group (-OH) of the acceptor with an α-linked bond [12, 17, 29]. Transglucosylation reactions of DGAS were evaluated using sucrose as a donor and nine flavones (apigenin, chrysin, 6,7-dihydroxyflavone, homoorientin, 7-hydroxyflavone, isorhoifolin, luteolin, luteolin-3′,7-diglucoside, and orientin) as acceptors (Fig 2) and the reaction mixtures were analyzed by HPLC to confirm the transglucosylated products (S1 Fig). No new peaks in the chromatograms were detected for the reaction mixtures of chrysin, 7-hydroxyflavone, luteolin-3′,7-diglucoside, and orientin by HPLC. Although there was variation in the reaction efficiency, a new peak was detected in chromatograms for five flavone reactants (luteolin, apigenin, 6,7-dihydroxyflavone, homoorientin, and isorhoifolin), suggesting that these five flavones could be used as acceptors for the production of α-flavone glucosides by DGAS (Table 1).

**Table 1:** DGAS acceptor specificity using various flavones.

Flavone compounds	Molecular weight	Hydroxyl group location	Conversion (%)	Reference
Baicalein	270.2	5,6,7	59.1	[10]
Apigenin	270.2	4',5,7	19.6 ± 5.0	In this study
Isorhoifolin (Apigenin-7-*O*-rutinoside)	578.5	4',5	1.8 ± 0.7	In this study
Luteolin	286.2	3',4',5,7	86.0 ± 5.0	In this study
Homoorientin (Luteolin-6-C-glucoside)	448.4	3',4',5,7	57.0 ± 1.4	In this study
Orientin (Luteolin-8-C-glycoside)	448.4	3',4',5,7	N.D.	In this study
Luteolin-3',7-diglucoside	610.5	3',4',5	N.D.	In this study
7-Hydroxyflavone	238.2	7	N.D.	In this study
Chrysin	254.2	5,7	N.D.	In this study
6,7-Dihydroxyflavone	254.2	6,7	56.0 ± 1.5	In this study

N.D., not determined.

A previous study has shown that α-BG can be successfully synthesized by DGAS [10]. However, the transglucosylation reaction has not been demonstrated using chrysin and 7-hydroxyflavone, with similar structures to that of baicalein (i.e., hydroxyl groups are present only in the A-ring). Interestingly, using 6,7-dihydroxyflavone, the transglucosylation reaction occurred, suggesting that DGAS could transfer glucose to the 6^th^ position of the hydroxyl group of the A-ring. There are differences in transglycosylation reactions depending on the number and site of hydroxyl groups in the phenol ring structure due to differences in their ion energy levels [30, 31]. The C6 hydroxyl site is glycosylated when there are no hydroxyl groups in the 4′C of the B-ring, e.g., in baicalein [9]. Nevertheless, 6-*O*-glycosylation is rarely observed because there are few hydroxyls in C6 and C8 of the A-ring in natural flavonoids [32]. Using apigenin and luteolin, in which a hydroxyl group is present in the B-ring of the flavone structure, α-flavone was synthesized by the transglucosylation reaction of DGAS. Since DGAS is limited with respect to the transfer of glucose to the C5 and C7 hydroxyl group positions at the A-ring, the synthesized apigenin glucoside was predicted to be apigenin-4'-*O*-α-d-glucopyranoside. A previous study has shown that DGAS could not transfer glucose to the rutinoside of the glycoside [19]. However, isorhoifolin, a glycoside with rutinoside and apigenin, was produced by the transglucosylation reaction. In addition, the transglucosylation efficacy was similar to that for apigenin in the aglycone form. Therefore, the isorhoifolin glycoside produced by DGAS was predicted to be isorhoifolin-4'-*O*-α-d-glucopyranoside. Notably, LG was synthesized with high efficiency by DGAS using luteolin as an acceptor. Orientin (luteolin-8-C-glycoside) and luteolin-3',7-diglucoside were not used as acceptors by DGAS. A transglucosylation reaction with a high molecular weight acceptor molecule may be less feasible owing to steric hindrance at the substrate binding site of the enzyme [31]. Luteolin has two hydroxyl groups at the 3' and 4'-positions of the B-ring compared to apigenin. Two LGs (luteolin-3'-*O*-α-d-glucopyranoside and luteolin-4'-*O*-α-d-glucopyranoside) have been synthesized by glucansucrase using sucrose and luteolin as a donor and acceptor, respectively [33, 34]. NPAS (ASase from *Neisseria polysaccharea***)** synthesized only luteolin-4'-*O*-α-d-glucopyranoside and produced LG with very low efficiency (7%). Thus, NPAS variants were developed with improved transglycosylation activity to increase LG conversion to 65% [34]. The acceptor specificity of ASases vary among microbial species [25, 29, 35, 36], and DGAS showed higher LG production than that of NPAS.

### Separation and identification of LG

**Fig 3:**
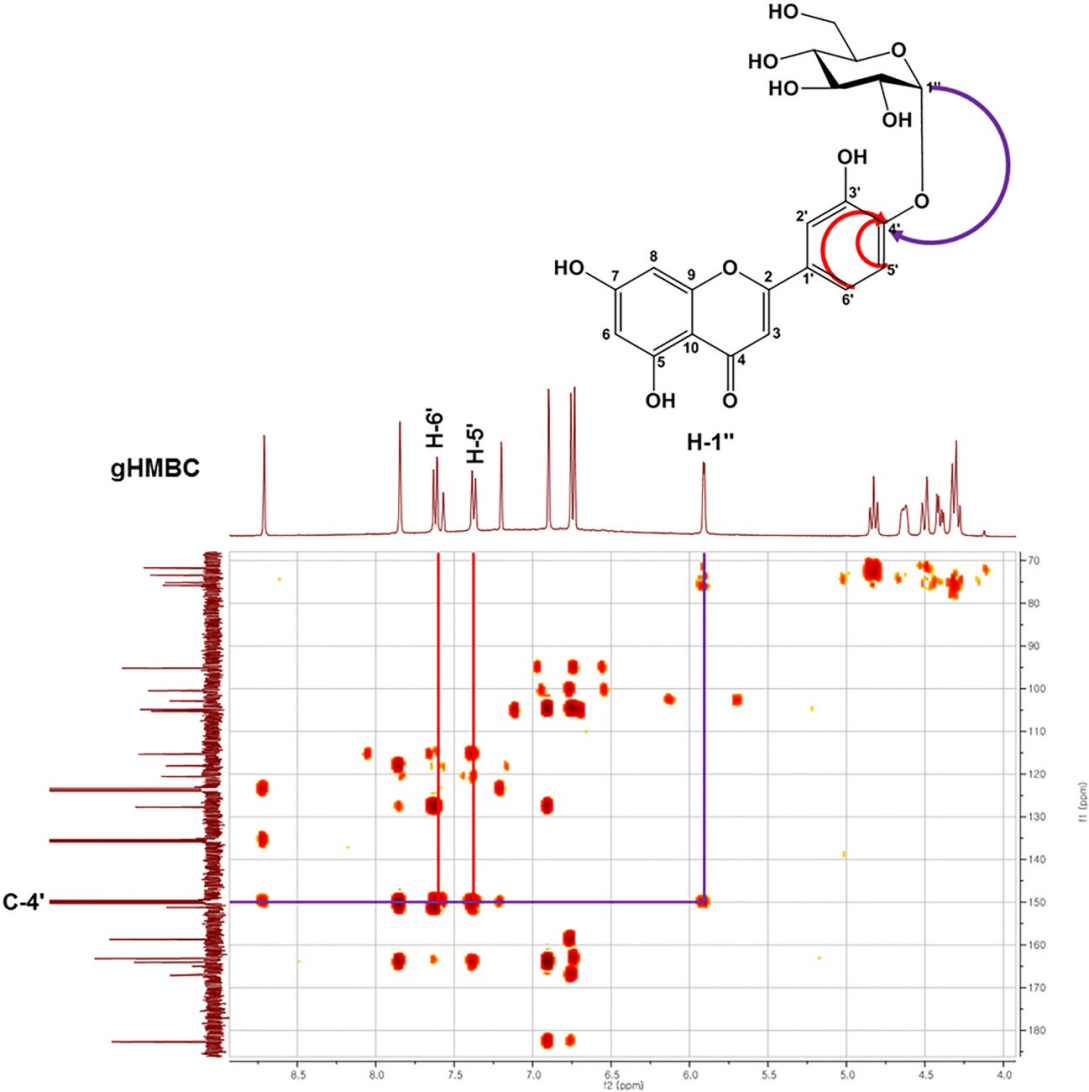
HMBC spectra of luteolin 4ʹ-*O*-α-D-glucopyranoside.

LG was separated and purified using semi-preparative HPLC systems (S2A Fig). The components of each fraction were identified by HPLC, and only fractions with pure LG were selected. The selected fractions were concentrated and freeze-dried. The purity was greater than 98% as confirmed by HPLC (S2B Fig), and the molecular weight of the purified LG was 447.07 *m/z* (M-H)^-^, which exactly matched the calculated molecular mass of LG. The ^13^C NMR spectrum displayed 21 carbon signals, indicating that the compound was a flavone monoglycoside, consistent with the ^13^C NMR spectrum for luteolin monoglucoside synthesized by glucansucrase [33, 34]. The ^1^H NMR spectrum showed a single, double acetal proton signal at δH 5.90 with a coupling constant of 3.6 Hz, indicating that the anomer carbon of glucose was α-based. In the heteronuclear multiple-bond correlation (HMBC) spectrum, the anomer proton signal (δH 5.90, H-1′′) showed a correlation with the oxygenated olefin quaternary carbon signal (δC 149.2, C-4′) (Fig 3), suggesting that the glycosidic bond was at C-4′. Therefore, The LG synthesized by DGAS was identified as luteolin-4′-*O*-α-D-glucopyranoside (α-LG) (Fig 3).

### Optimization of the bioconversion of luteolin to LG

**Fig 4:**
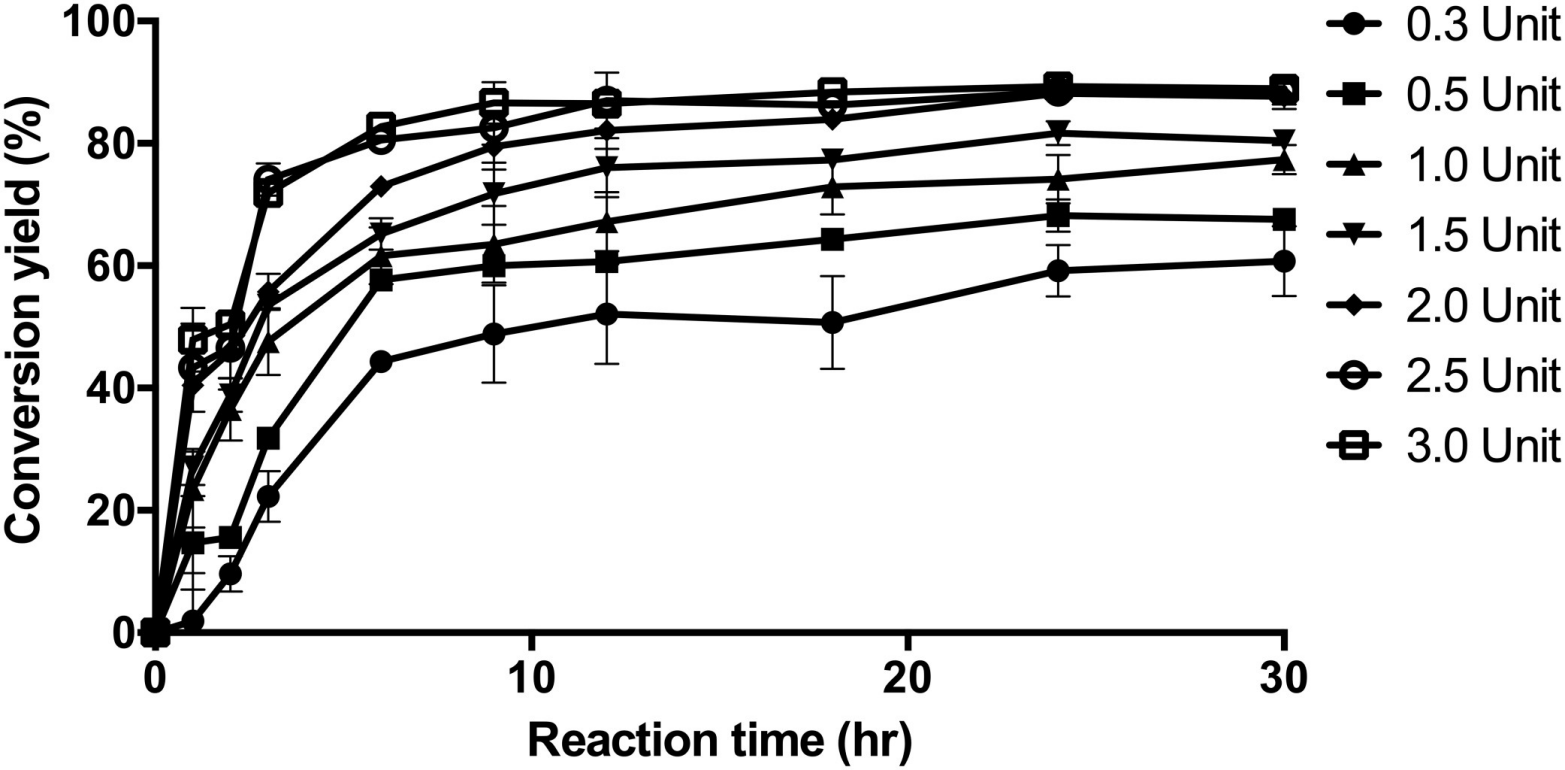
The conversion yield of 4ʹ-*O*-α-D-glucopyranoside synthesis reaction for various DGAS units. The units of DGAS were 0.3, 0.5, 1.0, 1.5, 2.0, 2.5, and 3.0.

Although α-LG has better water-solubility and oxidation stability than those of luteolin, its synthesis efficiency is less than 40% [33]. Recently, an α-LG synthesis efficiency of 70% was obtained using engineered NPAS, but it was not synthesized to a single component, as well as requiring a high sucrose concentration as a donor molecule [34]. DGAS showed a synthesis efficiency of greater than 70% for single α-LG in previously reported optimal reaction conditions (in 50 mM Tris-HCl buffer (pH 7) at 37°C) [17, 19, 37]. The conversion yield was evaluated according to DGAS units and reaction time with a 7:1 molar ratio of donor and acceptor molecules (Fig 4). The conversion rate increased significantly during the 6 h reaction period, and then the moderate conversion was observed for all DGAS units. The final conversion rate increased as the DGAS units increased, and there was no significant difference in the conversion rate of DGAS above 2 U after 18 h of reaction. The maximum yield of α-LG was 90%, which is the highest efficiency observed for glucansucrase [33, 34].

## Conclusions

Our results indicated that DGAS might transfer glucose to specific hydroxyl groups (the 6^th^ position in the A-ring and 4ʹ-position in the B-ring) of the flavone structure. In particular, DGAS used luteolin as an acceptor in flavones and synthesized luteolin-4'-*O*-α-D-glucopyranoside with high efficiency. Furthermore, conditions for synthesis efficiency of up to 86% were established by adjusting the enzyme concentration and reaction time.

## Supporting information

S1 Table Structural characterization of synthesized luteolin glucoside by DGAS. (DOCX)

S1 Fig HPLC analysis of the transglucosylation reaction by DGAS. Figures show the results of the analysis with various flavones and sucrose as the acceptor and donor, respectively. (Black line: reaction mixture without DGAS, Red line: reaction mixture with DGAS).
(DOCX)

S2 Fig Purified and mass analysis of synthesized luteolin glucoside by DGAS. FPLC chromatogram of luteolin reaction mixture with DGAS (A), HPLC /UV analysis of isolated LG fractions (B).
(DOCX)
