# Supplementary material for: Enzymatic synthesis of α-flavone glucoside via regioselective transglucosylation by amylosucrase from Deinococcus geothermalis
Source: PLoS One. 2018 Nov 19;13(11):e0207466. doi: 10.1371/journal.pone.0207466 (PMC6242681; doi:10.1371/journal.pone.0207466)
Supplement: S1 Table — (DOCX) [file pone.0207466.s001.docx]

**S1 Table. Structural characterization of synthesized luteolin glucoside by DGAS.**

| ^1^H-NMR (400 MHz, pyridine-*d*_5_, δ_H_) | 7.84 (1H, br.s, H-2'), 7.62 (1H, br. d, *J*=8.4 Hz, H-6'), 7.37 (1H, d, *J*=8.4 Hz, H-5'), 6.89 (1H, s, H-3), 6.75 (1H, d, *J*=1.2 Hz, H-8), 6.73 (1H, d, *J*=1.2 Hz, H-6), 5.90 (1H, d, *J*=3.2 Hz, H-1''), 4.82 (1H, dd, *J*=12.0, 5.2 Hz, H-6''a), 4.63 (1H, dd, *J*=12.0, 1.2 Hz, H-6''b), 4.49 (1H, dd, *J*=8.0, 8.0 Hz, H-3''), 4.40 (1H, dd, *J*=8.0, 8.0 Hz, H-4''), 4.31 (1H, dd, *J*=8.0, 3.2 Hz, H-2''), 4.29 (1H, dd, *J*=8.0, 8.0 Hz, H-5'') |
| --- | --- |
| ^13^C-NMR (100 MHz, pyridine-*d*_5_, δ_C_) | 181.8 (C-4), 166.2 (C-7), 163.1 (C-2), 162.3 (C-5), 157.8 (C-9), 150.3 (C-3'), 149.2 (C-4'), 126.8 (C-1'), 119.6 (C-6'), 117.2 (C-5'), 114.4 (C-2'), 104.4 (C-3), 103.9 (C-10), 101.9 (C-1''), 99.5 (C-6), 94.3 (C-8), 74.9 (C-5''), 74.2 (C-3''), 72.5 (C-2''), 70.8 (C-4''), 61.7 (C-6'') |
