# Supplementary material for: Enzymatic synthesis of α-flavone glucoside via regioselective transglucosylation by amylosucrase from Deinococcus geothermalis
Source: PLoS One. 2018 Nov 19;13(11):e0207466. doi: 10.1371/journal.pone.0207466 (PMC6242681; doi:10.1371/journal.pone.0207466)
Supplement: S1 Fig — Figures show the results of the analysis with various flavones and sucrose as the acceptor and donor, respectively. (Black line: reaction mixture without DGAS, Red line: reaction mixture with DGAS). (DOCX) [file pone.0207466.s002.docx]

**
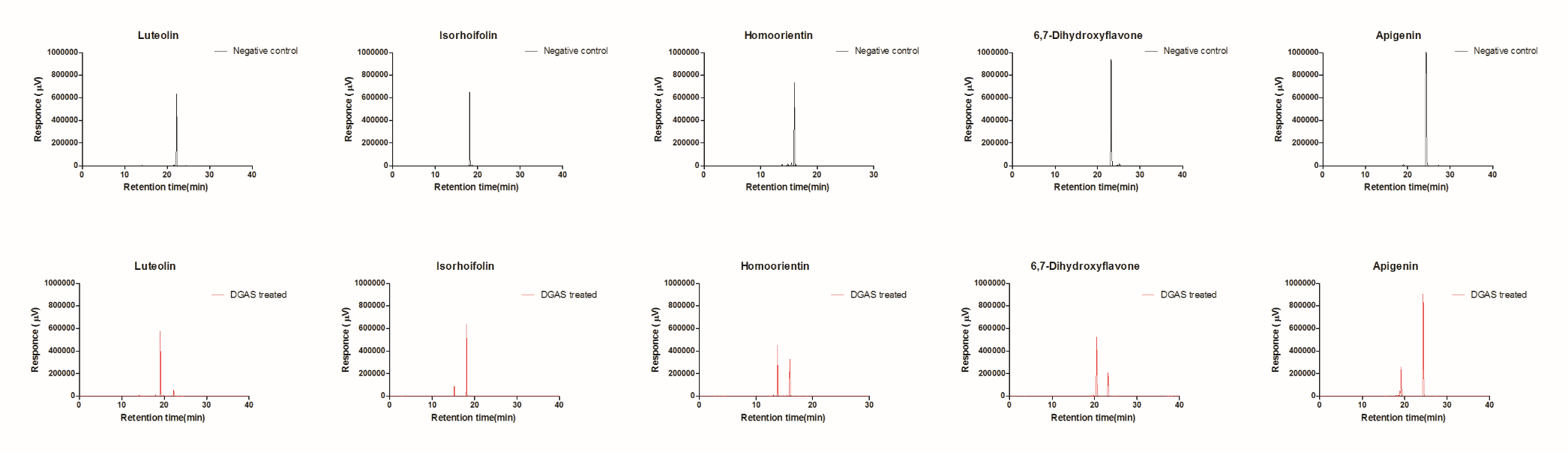
**

**S1** **Fig. HPLC analysis of the transglucoylation reaction by DGAS.** Figures shows the results of the analysis with various flavone and sucrose as the acceptor and donor, respectively. (Black line: reaction mixture without DGAS, Red line: reaction mixture with DGAS)
