# Supplementary material for: Enzymatic synthesis of α-flavone glucoside via regioselective transglucosylation by amylosucrase from Deinococcus geothermalis
Source: PLoS One. 2018 Nov 19;13(11):e0207466. doi: 10.1371/journal.pone.0207466 (PMC6242681; doi:10.1371/journal.pone.0207466)
Supplement: S2 Fig — FPLC chromatogram of luteolin reaction mixture with DGAS (A), HPLC /UV analysis of isolated LG fractions (B). (DOCX) [file pone.0207466.s003.docx]

**
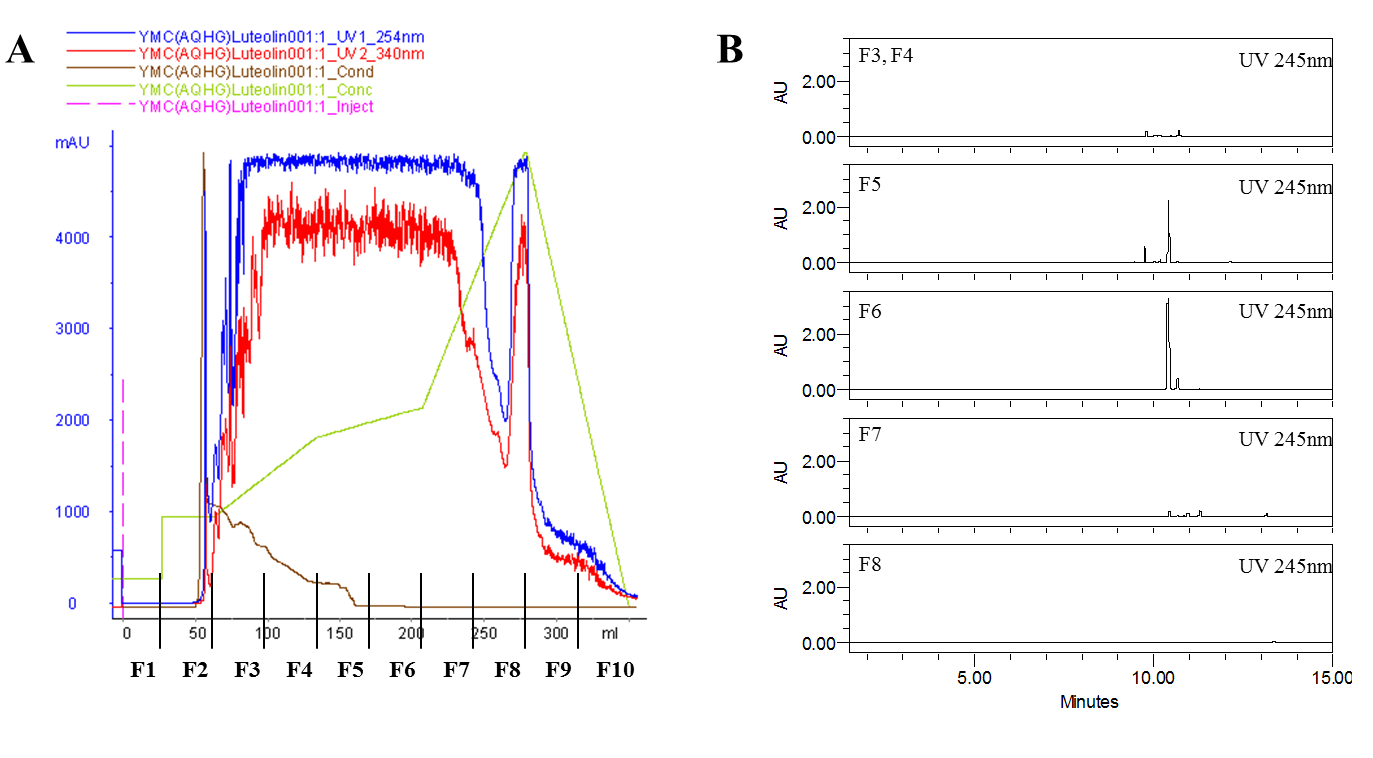
**

**S2 Fig. Purified and mass analysis of synthesized luteolin glucoside by DGAS.** FPLC chromatogram of luteolin reaction mixture with DGAS (A), HPLC /UV analysis of isolated LG fractions (B).
